# Reply to Troisi et al. Comment on “Cabrera-Aguas, M.; Watson, S.L. Updates in Diagnostic Imaging for Infectious Keratitis: A Review. *Diagnostics* 2023, *13*, 3358”

**DOI:** 10.3390/diagnostics15020171

**Published:** 2025-01-14

**Authors:** Maria Cabrera-Aguas, Stephanie L. Watson

**Affiliations:** 1 Sydney Medical School, Faculty of Medicine and Health, University of Sydney, Sydney, NSW 2000, Australia; stephanie.watson@sydney.edu.au; 2 Sydney Eye Hospital, Sydney, NSW 2000, Australia

We appreciate the interest of Troisi and his colleagues [1] in our article published in *Diagnostics* [2]. In this article, we aimed to review the corneal imaging diagnostic tests in current use for infectious keratitis and the rapid advancement of artificial intelligence (AI) methods to diagnose the types of infectious keratitis. We described the following diagnostic tools: slit-lamp microscopy and the use of digital cameras and smartphones, anterior segment optical coherence tomography, in vivo confocal microscopy, and the application of AI deep learning methods.

We sincerely appreciate the constructive comments. The cytological and microbiological examination of conjunctival and corneal scraping using scanning electron microscopy (SEM) has been reported for use in keratitis. The advantages of this diagnostic test include simple conjunctival collection, rapid analysis (within 72 h), high sensitivity and specificity, its use in a wide range of indications (allergic and autoimmune conditions and bacterial, viral, and parasitic keratitis), and the ability to reveal the morphological characteristics of the isolated organism [3,4]. Disadvantages include an operator-dependent technique, the difficulty of specimen preparation, an inability to detect viral organisms, the need for suitable equipment, and potential costs [3,4].

The first line in the diagnosis of infectious keratitis is still the Gram-stain and culture of corneal scrapings [5]. Advantages include the reporting of the antibiotic sensitivity and low cost, if the high capital and maintenance costs for MALDI-TOF are excluded [6]. Disadvantages include the turnaround times for the results of corneal scraping of within 48 h for bacteria and up to 10 days for slow-growing organisms; the need for trained laboratory staff to perform biochemical and phenotypic testing; the inability to distinguish contaminating microbes, viral agents, allergic, or autoimmune conditions; the results depending on the culture media used; and a low culture positivity rate (38–66%) [3,6,7].

Cytological evaluation using SEM seems promising as part of the diagnostic test pool for infectious keratitis. For example, Troisi et al. examined 65 patients with signs and symptoms of microbial keratitis with negative bacterial and fungal tests. These patients received antibiotic therapy for five days without improvement before undergoing the cytological evaluation of conjunctival scrapings of the superior tarsal conjunctiva by SEM. The causal organism was identified in 93% of the patients (61/65). The most common causal organisms identified were Candida (21), Acanthamoeba (21), and atypical organisms Mycoplasma (19), Chlamydia (7), and Mycobacteria (7) [3]. Similarly, in another study, Troisi et al. examined 102 eyes of 87 patients with the cytological examination of conjunctival scrapings of the superior tarsal conjunctiva using SEM. Organisms were isolated in 94/102 eyes (92%) by two independent operators with 99% operator agreement [8].

We agree with Troisi at et. that the cytological evaluation of conjunctival and corneal scraping using SEM is a potential method for the diagnosis of infectious keratitis. This test can be used along with the standard corneal scraping culture and other diagnostic imaging tests depending on the clinical history of the patient and the availability of tests at the health facility. We support the notion that an updated and comprehensive review of cytological evaluation using SEM should be included in future reviews of methods for the diagnosis of infectious keratitis [3].
